# Supplementary material for: Macroscopic photonic single crystals via seeded growth of DNA-coated colloids
Source: Nat Commun. 2023 Jul 15;14:4237. doi: 10.1038/s41467-023-39992-3 (PMC10349826; doi:10.1038/s41467-023-39992-3)
Supplement: Supplementary file 1 — Supplementary Information [file 41467_2023_39992_MOESM1_ESM.pdf]

SUPPLEMENTARY INFORMATION FOR "MACROSCOPIC PHOTONIC SINGLE CRYSTALS VIA SEEDED GROWTH OF DNA-COATED COLLOIDS"

SUPPLEMENTARY NOTE 1. COMPENSATING FOR PARTICLE WEAKENING DURING SEEDED GROWTH EXPERIMENTS

We notice a very slow, apparent 'weakening' of the DNA-mediated interactions during the seeded growth experiments, which we compensate for by gradually lowering the temperature. The evidence of the apparent weakening of the interactions is that the assembled crystals begin to melt after a few hours of growth at constant temperature. We attribute this effect to an interaction between the DNA-coated colloidal particles and the residual oil/surfactant that is left over from the broken emulsion, which we hypothesize interferes with the surface-grafted DNA molecules, thereby lowering the melting temperature of the suspension over time. We highlight that the weakening of the interactions is inconsistent with simply a reduction in the supersaturation due to the depletion of monomers, because it is physically impossible that a reduction in the supersaturation would lead to crystal melting. At the very most, the crystal and gas would reach equilibrium coexistence and growth would cease.

To offset the 'weakening' of the DNA-mediated interactions over time, we track the area of the same reference crystal continuously throughout the experiment and decrease the temperature if we see that the crystal begins to shrink. More specifically, after growth begins, if the area of this crystal decreases between two successive frames (i.e., after 10 min), a counter is incremented. Once this counter reaches two, the temperature is lowered by 0.05 °C. An example of the temperature trace over an entire experiment is shown in Suppl. Fig. 1, where the temperature is lowered by 0.15 °C over the course of roughly 14 h. We stress that reducing the temperature with time is simply to maintain a constant supersaturation and not to accelerate growth.

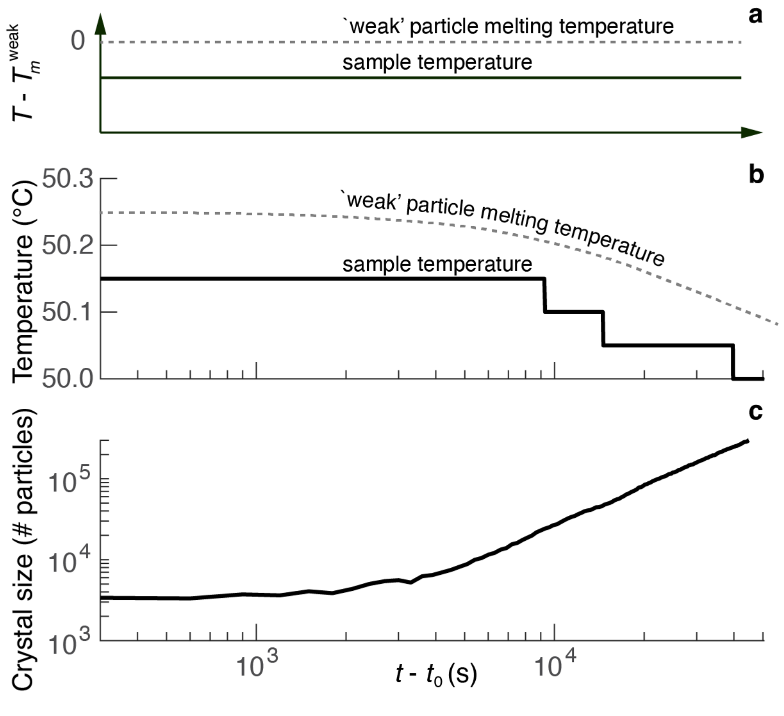

Supplementary Figure 1. **Overview of the seeded growth feedback-control scheme.** Plots showing the sample temperature relative to the weak particle melting temperature (**a**), the weak particle melting temperature and the sample temperature (**b**), and the size of the reference crystal (**c**) as a function of time. The sample temperature is decreased three times across the experiment keep the difference between the sample temperature and the melting temperature constant, thereby preventing the crystal from shrinking in size.

SUPPLEMENTARY NOTE 2. DATA ANALYSIS

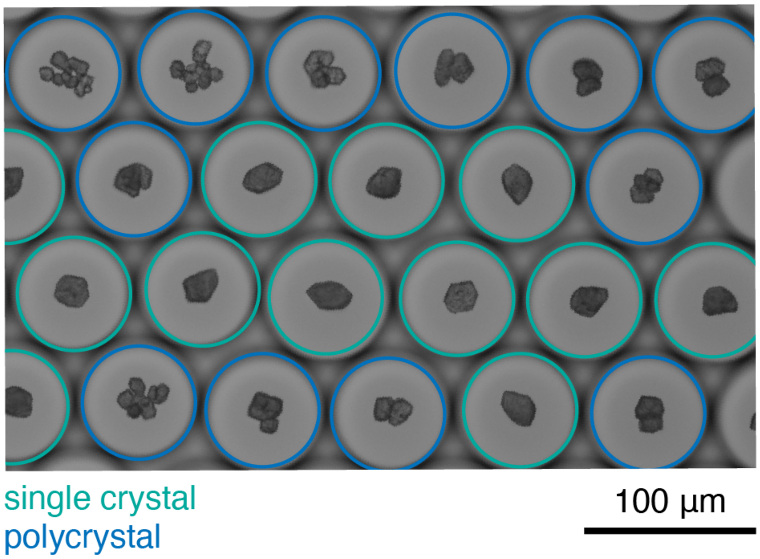

Supplementary Figure 2. **Quantifying single crystals in droplets.** An example image of crystals from a droplet temperature ramp experiment. Some droplets have single crystals (green), others have polycrystals (blue).

A. Determining the single crystal fraction

Single crystals are identified manually to compute the single crystal fraction. In brief, an image of an entire camera field of view is loaded into a standard computer paint program at a magnification that is sufficient to make out the details in the individual crystals (Suppl. Fig. 2. Single crystals or polycrystals are determined by eye by looking either for the presence of grain boundaries or for an irregularly shaped crystal as opposed to a well faceted crystal. Droplets that appear to contain a single crystal are marked with a red dot and droplets that appear to contain a polycrystal are marked with a blue dot. These images are saved and then analyzed via a MATLAB script that tallies the number of red dots, N_{r}, and blue dots, N_{b}, and then calculates the proportion of single crystals as $p_{1\mathrm{x}} = N_{\mathrm{r}}/(N_{\mathrm{r}} + N_{\mathrm{b}})$. Droplets that are out of focus or appear to have an anomalously low or high numbers of particles are excluded from the analysis.

B. Determining the crystal size

Seeded growth. We determine the number of particles per crystal for our seeded growth experiments using an empirical calibration curve based on the projected area of the single crystals. For each of the distinct droplet volumes used in the temperature-ramp experiments (Fig. 2c-d in the main text), we measure the crystal area of a large population of single crystals. For each droplet volume, we know the total number of particles inside the droplet since we know the total particle volume fraction: $N_{\mathrm{drop}} = V_{\mathrm{drop}}\rho_0$, where ρ_0 is the known concentration of particles used in microfluidic drop making and V_{drop} is the measured volume of a droplet in the given experiment. Because all of the particles are incorporated into the crystal phase by the end of the temperature-ramp experiment, we know that $N_{\mathrm{crystal}} = N_{\mathrm{drop}}$.

We plot the mean crystal area with respect to the number of particles inside the droplet and find that it follows a power law (Suppl. Fig. 3). A fit of a power law to our data shows that the number of 600-nm-diameter particles per crystal follows the scaling relation $N_{\mathrm{crystal}} = 3.28A_{\mathrm{crystal}}^{1.4}$, where A_{crystal} is the crystal area in $\mu\mathrm{m}^2$. We then use this expression to infer the number of particles per crystal for our seeded growth experiments (see Fig. 3 in the main text). Note that the expected power-law dependence would be $N_{\mathrm{crystal}} \propto A_{\mathrm{crystal}}^{3/2}$. We hypothesize that the small difference is due to the fact that the crystals are slightly

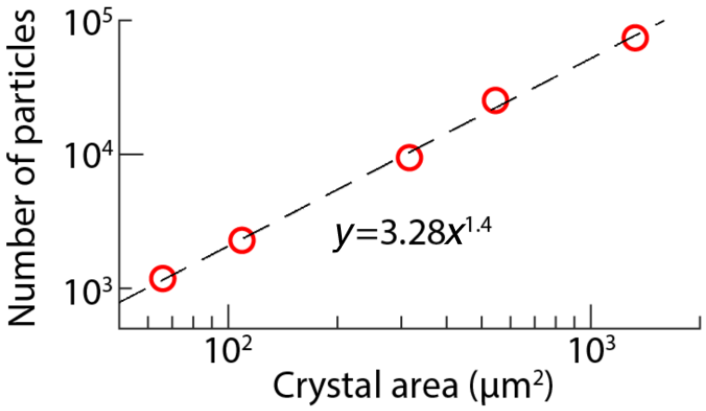

Supplementary Figure 3. **Calibration of crystal size versus crystal area.** The number of particles in a crystal is extrapolated from the projected area of the crystal via the empirical expression $N_{\mathrm{crystal}} = 3.28A_{\mathrm{crystal}}^{1.4}$.

more dense than water and thus settle to the bottom of the sample chamber once they are sufficiently large.

Comparing to literature. Because we cannot use the same calibration curve to estimate the volumes of crystals made from colloidal particles of differing diameters reported in the literature, we estimate the crystal sizes using a much more approximate method. In particular, in Fig. 4b, we estimate the crystal sizes from our two-step method by taking the cube of the linear dimension of the crystal s relative to the interparticle spacing. We expect that this estimate is an upper bound of the crystal volume, as evidenced by our calibration method described above.

Suppl. Fig. 4 shows estimates of the crystal sizes collected from a review of the literature since 2011. The values for the points, as well as the references, can be found in Suppl. Table 2. Because we ultimately want to make comparisons between similar experimental systems, we focus only on articles in which: (i) the interactions between particles are mediated by DNA hybridization between grafted strands; (ii) the particles are roughly spherical and have isotropic interactions; (iii) an image of an assembled crystal is shown in the main text; and (iv) the image has either clear faceting, denoting the single crystalline nature of the habit, or shows single particle resolution of a single crystalline domain. For crystals that follow these criteria we measure the longest linear dimension of a single domain. For particle diameters that are below 100 nm, the ssDNA brush can increase the lattice spacing of the crystal by a substantial fraction relative to the particle diameter. Therefore, when possible, the lattice spacing is inferred from either the spacing of particles in the images or from the first peak in any provided small angle X-ray scattering (SAXS) data. We then compute the crystal size as the cube of the longest linear dimension divided by the lattice spacing. Again, since we do not consider the packing fraction of the different crystal types that form, these data denote an upper bound on the size of the crystalline assemblies in the literature.

Our survey of the literature shows that the size of the largest crystals assembled from DNA-coated particles decreases with increasing particle diameter. Specifically, we plot the largest estimated crystal sizes for particle diameters of 10 nm, 15 nm, 20 nm, 60 nm, 400 nm, 600 nm, and 1000 nm to cover the typical range of particle sizes from the nanometer to the micrometer scales (see Fig. 4b). We hypothesize that the trend of decreasing crystal size with increasing particle diameter results primarily from changes in nucleation behavior that depend on the relative range of the DNA-mediated attraction between particles, which is roughly equal to the ratio of the end-to-end length of the grafted DNA molecules to the diameter of the particles. Because the end-to-end length of the DNA molecules grafted to nanoparticles and microparticles are typically comparable (order ten nanometers), the relative range of attraction decreases from about 100% of the particle diameter to 1% of the particle diameter going from 10-nm- to 1000-nm-diameter particles (i.e., roughly the range of diameters shown in Fig. 4b).

Supporting this hypothesis, previous studies of colloid–polymer mixtures have shown that the range of the interaction potential is critical in determining the topology of the phase diagram and the nature of the crystallization process [1]. For example, while such systems show gas-liquid-crystal coexistence for sufficiently long-range attractions, shortening the relative range of attraction causes the fluid–fluid transition to become

metastable [2]. These changes to the phase behavior are accompanied by significant changes in nucleation. For example, depending on the presence of a stable liquid phase or the proximity of a metastable fluid–fluid critical point, it is possible for crystallization to proceed via a two-step pathway, in which a fluid droplet forms first and then the crystal nucleates from within that droplet [3]. The high density of the droplet is predicted to increase the crystal nucleation rate by lowering the surface tension. The high density of the fluid layer surrounding a crystallite also modifies the crystal growth rate by increasing the local concentration. As the relative range of attraction decreases, the accessibility of this two-step pathway diminishes. Although there is at present no direct experimental evidence that similar changes to the phase behavior and phase-transformation kinetics occur for systems of DNA-coated colloids, we note that the observed trend of decreasing crystal size (and increasing difficulty in controlling nucleation) with increasing particle size is in line with our expectations based on the colloid–polymer literature. We further note that decreasing the relative range of attraction is also likely to suppress surface diffusion, which tends to slow the growth of small crystallites [4] and may hamper the annealing of multi-domain crystals.

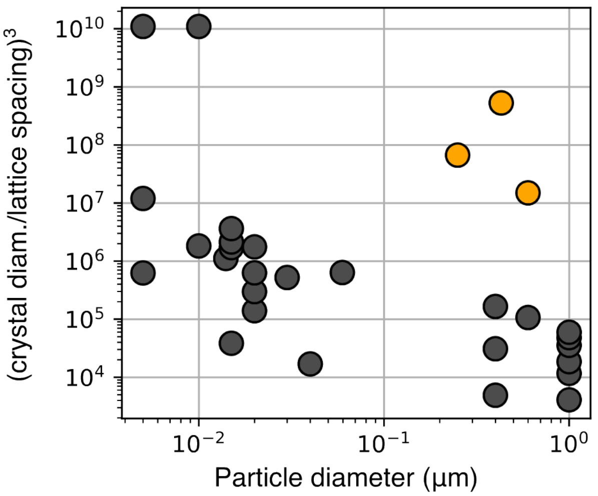

Supplementary Figure 4. **Review of reported single crystals.** An overview of the number of particles in various crystals reported in the literature for different particle sizes. These values are provided in Suppl. Table 2 at the end of the SI. Orange points are from this work.

C. Determining the start of growth for the seeded growth experiments

The seeded growth experiments are performed by starting at a temperature above the melting temperature of the 'weak' particles, but below the melting temperature of the seeds, and then lowering the temperature slowly until the crystals start growing. The start time of crystal growth t_0 is therefore determined by the frame at which the reference crystal first reaches a value 10% larger than its initial size.

SUPPLEMENTARY NOTE 3. SOLVING THE CRYSTAL STRUCTURES AND CRYSTAL HABITS

In the main text we show the crystallization and seeded growth of single-crystalline assemblies of three different binary mixtures of same-sized particles with diameters of 600 nm, 430 nm, and 250 nm. The following section describes the quantitative pipeline that we developed to characterize their 3D crystal structures, including both the lattice structure and the crystal habit.

A. An overview of the binary body-centered tetragonal crystal structure

All three crystal structures that we find can be classified within a binary version of the body-centered tetragonal (BCT) crystal structure. The binary BCT structure is characterized by having primitive vectors of $a_x\hat{x}$, $a_y\hat{y}$, and $a_z\hat{z}$, with the restriction that $a_x = a_y$ and the two sublattices of the binary mixture displaced by a vector $(a_x\hat{x} + a_y\hat{y} + a_z\hat{z})/2$, as shown in Suppl. Fig. 5a.

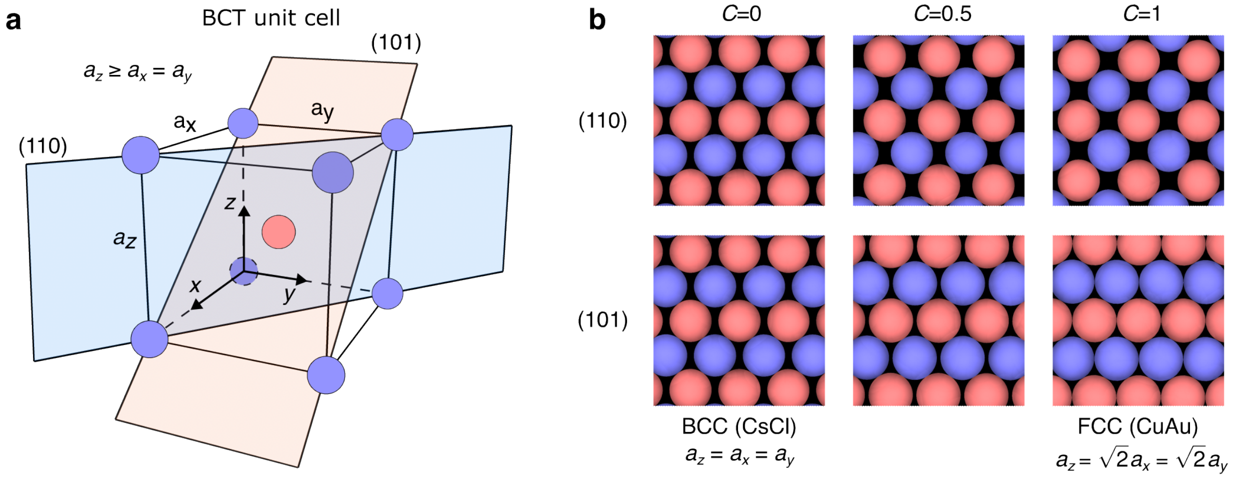

Supplementary Figure 5. **BCT crystal structure and facets.** (**a**) An illustration of a BCT unit cell. Note that with this definition of the BCT structure, we consider the vertical lattice spacing, a_z, to be greater than or equal to the other lattice spacings, a_x or a_y. The facets with the lowest surface energies for this structure are the (110) and (101) planes, shown by the blue and red planes, respectively. (**b**) Images of the (110) and (101) facets for different BCT configurations of C=0, 0.5, 1.0. Note that when $C = 0$, the BCT lattice is the same as BCC-CsCl, and when $C = 1$, the BCT lattice is the same as CuAu.

The class of binary BCT crystal structures has within it crystals that are isostructural to CsCl and CuAu. When $a_z = a_x = a_y$, we recover a binary simple cubic lattice, isostructual to CsCl, and when $a_z = \sqrt{2}a_x = \sqrt{2}a_y$, the lattice is isostructural to the CuAu lattice. To clearly see these two limits we show the (110) and (101) planes of the binary BCT lattice in Suppl. Fig. 5b on the left and right sides. Note that the (110) and (101) binary BCT planes are the same as the CsCl (110) and (101) planes (left), while in the other limit the binary BCT (110) is the same as the CuAu (100) plane and the binary BCT (101) plane is the same as the CuAu (111) plane. We choose only to show the (110) and (101) planes of the binary BCT structure since these are the lowest-surface-energy facets and are the mostly like to be present in the facets of crystals that form. This note will be described in more detail in the Suppl. Note 2B.

To characterize the BCT structures that lie between CsCl and CuAu, we introduce a linear transformation for the magnitudes of a_x and a_z,

$$a_x = a_y = \frac{2}{\sqrt{3}}a_0(1-C) + a_0C \tag{1}$$

$$a_z = \frac{2}{\sqrt{3}}a_0(1-C) + \sqrt{2}a_0C, \tag{2}$$

where a_0 is the spacing between the two particle types and C is a scalar. When $C = 0$, we recover CsCl, and when $C = 1$, we recover CuAu. As an example, we show the (110) and (101) planes for $C = 0.5$ in the middle column of Suppl. Fig. 5b. We note that if a_0 remains fixed, this transformation preserves each particle having eight nearest-neighbor contacts of the other particle type.

B. A quantitative method to characterize the 3D crystal structure

We develop a quantitative pipeline based on laser-scanning confocal microscopy, image analysis, and crystallography to determine the 3D crystal structures of the three different crystal types that we explore in

experiment. The pipeline is as follows: (i) collect images of the crystal facets that have sedimented during growth; (ii) find particle positions for both A and B particles using image analysis and compute their radial distribution functions, $G_{\mathrm{AB}}(r)$ and $G_{\mathrm{AA}}(r)$ (note that we only use $G_{\mathrm{AA}}(r)$ when one of the particles cannot be resolved); (iii) generate a look-up table of model $\tilde{G}(r)$ for potential crystalline facets (from here on $\tilde{G}$ will denote model functions); and (iv) compare the experimental $G(r)$ against all model $\tilde{G}(r)$ to find the closest match. As a complementary technique, we also take a scan orthogonal to the exposed facet to look at the crystalline order in the third dimension. This complementary method is described in Suppl. Note 2C.

We image the crystals with a Leica SP8 laser-scanning confocal microscope. Since our crystals are composed of two particle species, we independently dye each particle type, one with Pyrromethane 546 and one with Nile Red. We then take a two-color acquisition to capture the particle locations and types for a given crystallographic plane, as shown in Suppl. Fig. 6a. The crystal facet images are then analyzed to find particle positions [5]. In brief, the images are split into separate channels corresponding to the two particle types in the crystal; they are convolved with a Gaussian to reduce the noise; a local thresholding algorithm is used to isolate the center of particles and to compensate for any large-scale variations in image intensity; and lastly we compute the centroids to find the positions of all the particles in the facet. The positions of the particles are then used to calculate the experimental radial distribution function, $G(r)$ [6].

To create the reference $\tilde{G}(r)$ data, we identify relevant facets of proposed crystal structures that may show up in experiment. We then generate position data of A and B particles for those facets given a proposed crystal structure, as shown for an example model facet in Suppl. Fig. 6a. In our experiments, we primarily see the (110) and (101) planes of body-centered tetragonal (BCT) structures described in Suppl. Note 2B. Once the particle positions are defined, we calculate corresponding $G(r)$ data sets. In our lookup table we included the (100), (110), (111), and (210) facets of binary BCC (CsCl) and FCC (CuAu) structures, as well as the (110), (101) facets of BCT structures with C-values ranging from 0 to 1 in steps of 0.05. Other facets, such as the (100) or (111) facets of BCT, were excluded because we do not observe facets that contain only a single particle type.

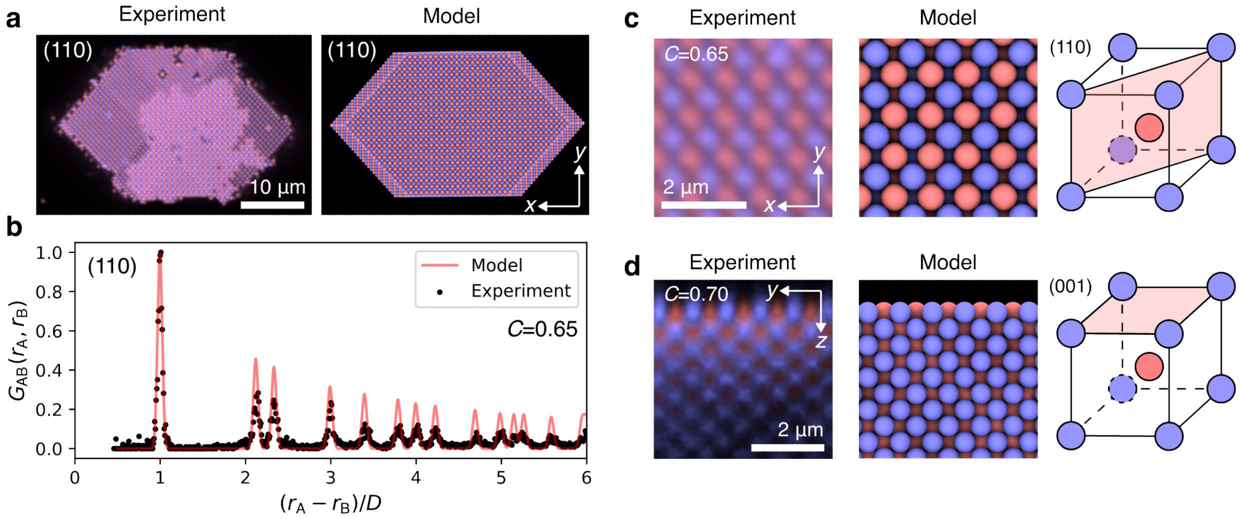

Supplementary Figure 6. **600-nm-diameter particle crystal characterization.** (**a**) A confocal image (left) of the presented facet of one crystal formed from a binary mixture of 600-nm-diameter particles as compared to the best-fit model (right). (**b**) The pair correlation function for unlike particle species showing both experimental data (points) from the image in (**a**) and a best-fit model from a BCT (110) facet with C=0.65. (**c**) Zoom in of the facet of the image in (**a**) along with a zoom in of the corresponding model BCT (110) facet. The facet is denoted by the red plane in the unit cell illustration. (**d**) A z-slice confocal image of a different crystal with BCT C=0.70 with a corresponding model slice. The facet is denoted by the red pane in the unit cell illustration. Note that the experimental image comes from a projection of four layers of the crystal as described in Suppl. Note 2C.

Next, we compare the experimental and model $G(r)$ data to identify the best match. In order to make this comparison, we need to introduce noise into our model data that is comparable to the noise in our experimental data. We fit the $G(r)$ peak that is closest to $(r_\mathrm{A} - r_\mathrm{B})/D = 1$ (2 for $G_{\mathrm{AA}}(r)$) with a Gaussian

and extract the mean and standard deviation. The mean is used to get the true lattice spacing of the crystal and to rescale the experimental $G(r)$ to have a peak at $(r_\mathrm{A} - r_\mathrm{B})/D$ at 1 (2 for $G_\mathrm{AA}(r)$). We then convolve the model data with a Gaussian that has the same standard deviation as the experiment. Both sets of data are then normalized to set the amplitude of the first peak to one. This normalization sets a reference point from which we can compare various model facets to our experiment. For a given model $\tilde{G}(r)$, we compute the sum of the squared residuals between the model and the experimental data, $\sum_i (G_(r_i) - \tilde{G}(r_i))^2$. We take the model with the smallest sum of the squared residuals as the best fit crystal structure to the experimental image. An example best fit is shown for 600-nm-diameter particles in Suppl. Fig. 6b.

A similar analysis can also be performed for internal crystal planes, as described in Suppl. Note 2B By taking a vertical scan through the crystals, we can observe the internal order and subsequently fit particle positions to find a best fit crystal structure. An example of such a plane for the 600-nm-diameter particles in shown in Suppl. Fig. 6d with a corresponding model plane next to it.

Example results of this pipeline for crystals composed of 430-nm and 250-nm particles are shown in Suppl. Fig. 7 and 8. We note that due to the size of the 250-nm particles, we were unable to resolve the Nile Red-dyed particles because they are below the diffraction limit as a result of their longer-wavelength emission, so only the Pyrromethane 546-dyed particles are used to compute $G_\mathrm{AA}(r)$.

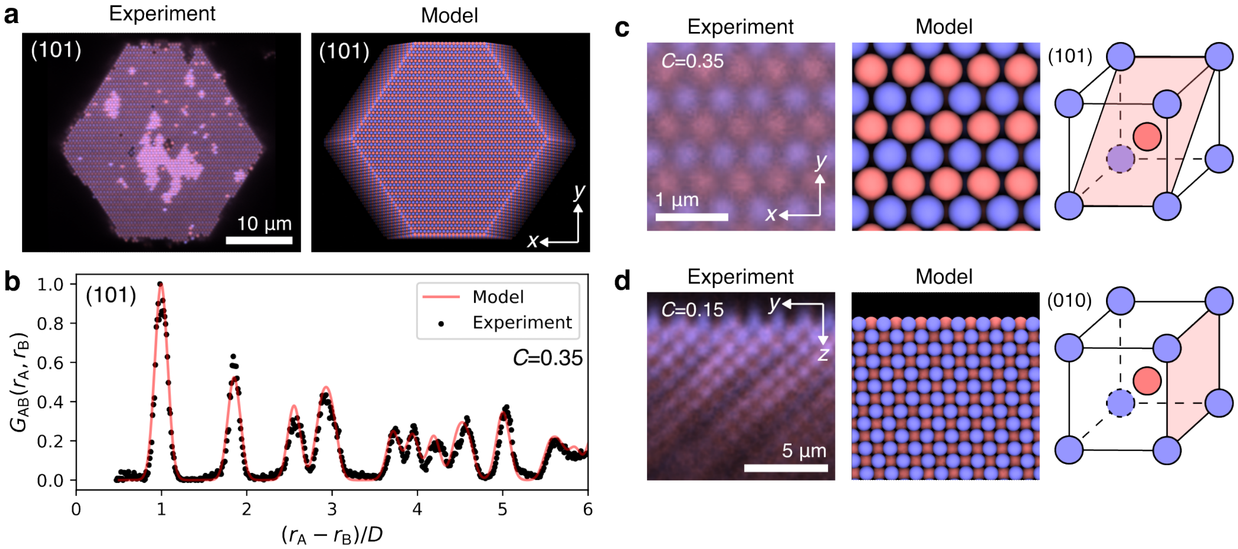

Supplementary Figure 7. **430-nm-diameter particle crystal characterization.** (**a**) A confocal image (left) of the presented facet of one crystal formed from a binary mixture of 430-nm-diameter particles as compared to the best-fit model crystal (right). (**b**) The pair correlation function for unlike particles showing both experimental data (points) from the image in (**a**) and a best fit model from a BCT (101) facet with C=0.35. (**c**) Zoom in of the facet of the image in (**a**) along with a zoom in of the corresponding model BCT (110) facet. The facet is denoted by the red plane in the unit cell illustration. (**d**) A z-slice confocal image of a different crystal with BCT C=0.15 with a corresponding model slice. The facet is denoted by the red plane in the unit cell illustration. Note that the experimental image comes from a projection of four layers of the crystal as described in Suppl. Note 2C.

C. Imaging the internal crystal structure

We use laser-scanning confocal microscopy to image several layers into the crystal to check the crystalline order of our structures in the third dimension. We first take an x-y scan to image the presented facet of the crystal. We then record an x-z scan to visualize the interior of the crystal. Because there is an index mismatch between the particles (roughly $n = 1.59$) and the solvent (roughly $n = 1.33$), we are only able to image ten or so layers and the image quality is reduced. To increase the contrast of the particles in the x-z image, we compute the variance of x-z slices over four particle diameters in the y direction. We intentionally choose either the (010) plane or the (001) plane, which both have alternating layers of a single particle type

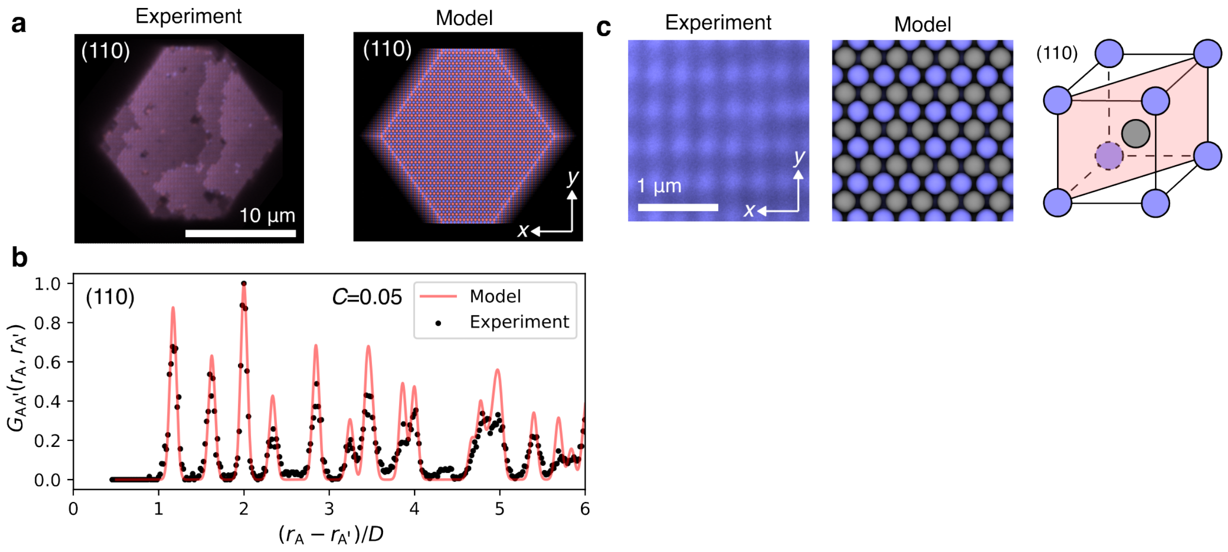

Supplementary Figure 8. **250-nm-diameter particle crystal characterization.** (**a**) A confocal image (left) of the presented facet of one crystal formed from a binary mixture of 250-nm-diameter particles as compared to the best-fit model crystal (right). (**b**) The pair correlation function for like particles showing both experimental data (points) from the image in (**a**) and a best fit model from a BCT (110) facet with C=0.05. Because we were unable to resolve the red particles at this particle size, they were not used for the analysis of the lattice structure. (**c**) Zoom in of the blue particles on the facet of the image in (**a**) along with a zoom in of the corresponding model BCT (110) facet. The facet is denoted by the red plane in the unit cell illustration. Note that the experimental image comes from a projection of four layers of the crystal as described in Suppl. Note 2C.

(i.e., the first plane is all A, the second plane is all B, and so forth.) These planes also have the property that subsequent layers of the same particle type are identical, with no shift in particle positions. Finally, we compute the $G_{\mathrm{AB}}(r)$ for the x-y image and the x-z image, and find the best fit BCT model for each. Importantly, we find consistent crystal structures for both planes for every crystal we tested. Exemplary images of these two orthogonal planes are shown in Suppl. Fig. 9a–c for the 430-nm-diameter and 600-nm-diameter particle crystals. Due to the width of the point spread function of our confocal microscope, the 250-nm-diameter particles could not be resolved in the z-direction. These measurements into the bulk of the crystals illustrate their single crystalline nature.

D. Characterizing the ensembles of crystals

To check whether or not all of the crystals formed within a single experiment have the same crystal type, we use our quantitative pipeline to characterize dozens of single crystals for each particle size. The first step in this process is to collect images of 50–100 different crystals assembled within a single crystallization experiment. To facilitate our automated crystal detection scheme described above, we only consider crystals that sit flat on the lower coverslip surface. Once the images are collected, we pass them through the pipeline described in Suppl. Note 2B as a single batch. After all images are analyzed, we check that both the particle finding and $G(r)$ fitting are satisfactory, and discard any images that have too few identified particles.

We find that all of the crystals within the ensemble have the same lattice type and compositional order for each particle size. Suppl. Fig. 10A shows histograms of the best fit BCT characterization of the structures for the collected crystal images for particle diameters of 600-nm (bottom), 430-nm (middle), and 250-nm (top). For each particle type, we find consistent BCT structures for all of the crystals within the ensemble. For example, the 250-nm-diameter particles exhibit a CsCl symmetry and compositional order with mean and median values of $C = 0.02 \pm 0.03$ and $C = 0$. The 600-nm-diameter particles show a BCT structure with mean and median values of $C = 0.65 \pm 0.09$ and $C = 0.65$, which is nearly indistinguishable from the CuAu structure without careful quantitative analysis. The 430-nm-diameter particles yield an intermediate BCT

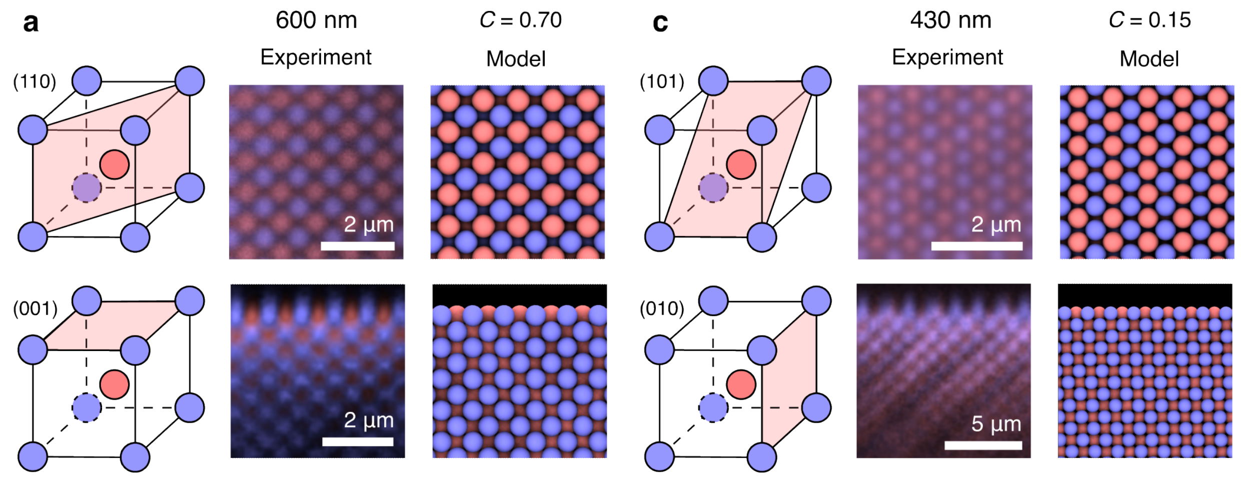

Supplementary Figure 9. **Determination of 3D bulk crystallinity.** Confocal images, along with the corresponding best-fit model images, for the presented facets (top), as well as a vertical slice into the crystal (bottom) for: (**a**) a 600-nm-diameter particle crystal and (**b**) a 430-nm-diameter particle crystal. Each pair of images comes from the same crystal. Model images are generated from measuring and fitting the $G(r)$ to different BCT structures. In both cases, a single BCT structure describes both the (110) and (001) crystal planes. Note that for both (**a**) and (**b**) the experimental images for the (001) and (010) planes come from a projection of four layers of the crystal.

structure with mean and median values of $C = 0.18 \pm 0.08$ and $C = 0.15$. For reference, Suppl. Fig. 10b shows the arrangement of particles on the most prominent facets for different BCT characterizations.

We hypothesize that these different crystal structures form due to a balance of specific attraction between 'unlike' particles due to DNA hybridization and nonspecific attraction between both 'unlike' and 'like' particles due to van der Waals attraction. A recent report from Pine and co-workers shows that polystyrene colloids coated with DNA in the same manner that we use in this article exhibit a weak, nonspecific attraction with an interaction strength of roughly 1–2 k_BT [7]. This weak interparticle attraction could stabilize the like–like contacts that are present in CuAu and absent in CsCl. Pine and co-workers also show that the range of this nonspecific attraction extends beyond the DNA-mediated potential minimum, which could allow the 'like' particles to sit at a slightly greater distance from one another as compared to the unlike particles, again as we see in our BCT crystal structures. Furthermore, theoretical models of the van der Waals interaction predict that the strength of attraction is proportional to the particle diameter [7]. Therefore, we would expect the nonspecific attraction to be weakest for the smallest particle sizes that we explore, which is consistent with our observation that the smallest particles form CsCl (which has no like–like particle contacts).

E. Understanding the crystal habits

Beyond the symmetry and compositional order of the crystal lattices, we also characterize and understand the crystal habits (i.e., the geometrical shapes of the single crystals). We hypothesize that the crystal habits that we observe are consistent with expectations from the Wulff construction, modified to account for the fact that crystal growth happens next to a flat surface.

Comparing crystal habits with the equilibrium Wulff construction. The Wulff construction is a method to determine the equilibrium shape of crystal structures that form [8–10]. Since different crystal planes have different surface energies, the crystal habit that minimizes the total surface energy per volume in general involves a complex combination of many crystallographic planes. The Wulff construction is an algorithmic method to predict the shape that minimizes the surface energy, assuming that the bounding surface comprises only allowed crystal planes. To construct this shape, one places crystal planes around an origin point so that the minimum distance from the point to the plane is proportional to the surface energy density of that plane. The minimum enclosed volume of all of these planes is the Wulff construction. In essence, this approach creates a geometry where the exposed planes are the low-surface-energy planes.

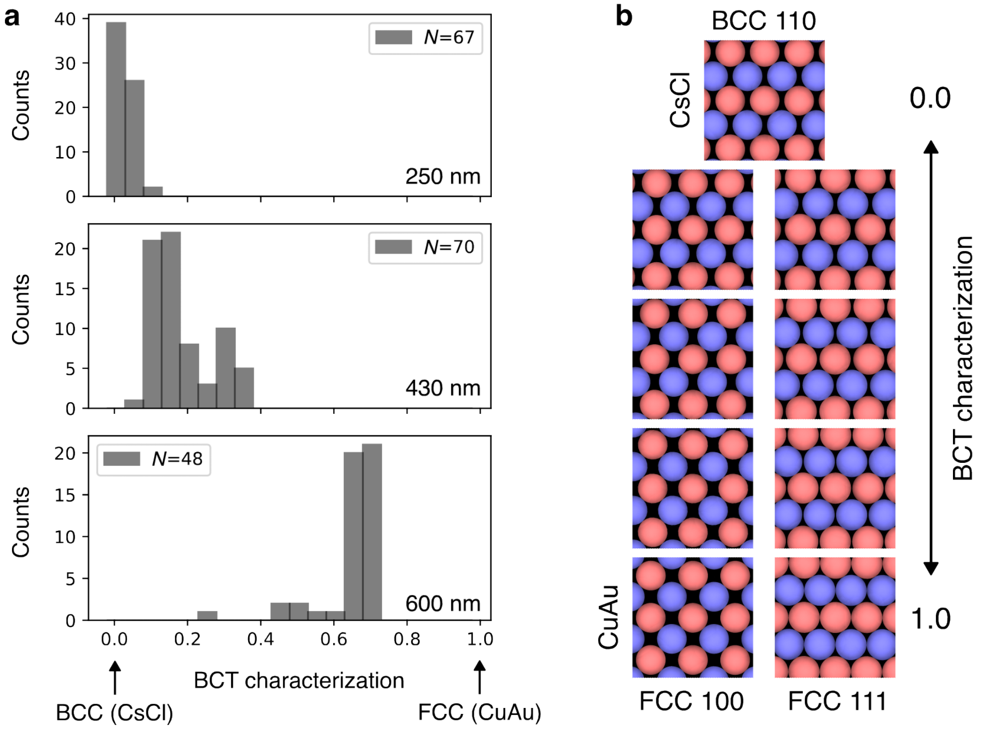

Supplementary Figure 10. **Distributions of crystal types for varying particle sizes.** (**a**) Histograms of measured C-values for many crystals from each of the three different-sized particles. (**b**) Close-up of model facets for different C-values that characterize the BCT structure of the crystals. C values of 0, 0.25, 0.5, 0.75, and 1.0 are shown in descending order.

Using the Wulff construction, we create model crystals based on estimates of the relative surface-energy densities of the binary BCT crystal structure. We estimate the surface energy density by considering the unit cell of a crystal plane and computing the ratio of the number of missing bonds to the area of the unit cell; the surface energy densities for different planes are rescaled by the lowest-surface-energy density plane. Such a Wulff construction is shown in Suppl. Fig. 11a for a BCT crystal with $C = 0.5$. For C-values between 0 and 1, the $(\pm 1 \pm 10)$, $(\pm 10 \pm 1)$, and $(0 \pm 1 \pm 1)$ planes have the lowest surface energies and have Wulff shapes that are distorted rhombic dodecahedra, which are stretched along the z-axis.

Accounting for interactions with the coverslip during late-stage growth. However, when we look at the (110) or (101) facets of these shapes, we find that they always have four sides, whereas the crystal facets that we find in experiment always have six sides. We hypothesize that this difference comes from the proximity of the coverslip and its effect on late-stage growth. To test this hypothesis, we carry out an analysis based on the Winterbottom construction [11], which we discuss next.

In the main text, we describe how we grow crystals either in droplets or in a bulk sample. In both of these cases, as the crystals increase in mass, they settle either at the bottom of the droplet, or against the bottom coverslip of the sample chamber. When this occurs, the crystal will preferentially grow from its exposed sides. This situation is reminiscent of the Winterbottom construction, where a crystal nucleates and grows preferentially from a surface [11, 12]. In our case, the substrate-facet surface energy is not relevant because the particles do not actually adhere to the interface.

To emulate the Winterbottom construction, we can take a crystal generated by the Wulff construction and pass a plane through it that we imagine being parallel to the substrate. Suppl. Fig. 11b shows an example with planes slicing through the (110) and (101) facets. This additional plane mimics the surface that the crystal is resting upon, so we reduce the crystal by removing all particles beneath the plane. If we perform this Winterbottom reduction for the (110) or (101) facets and look at the reduced face, we see they now have six sides. On each of these facets there are two distinct angles, α and β, which can therefore be used as another way to quantify the BCT structure of the crystal, since these angles change monotonically with C.

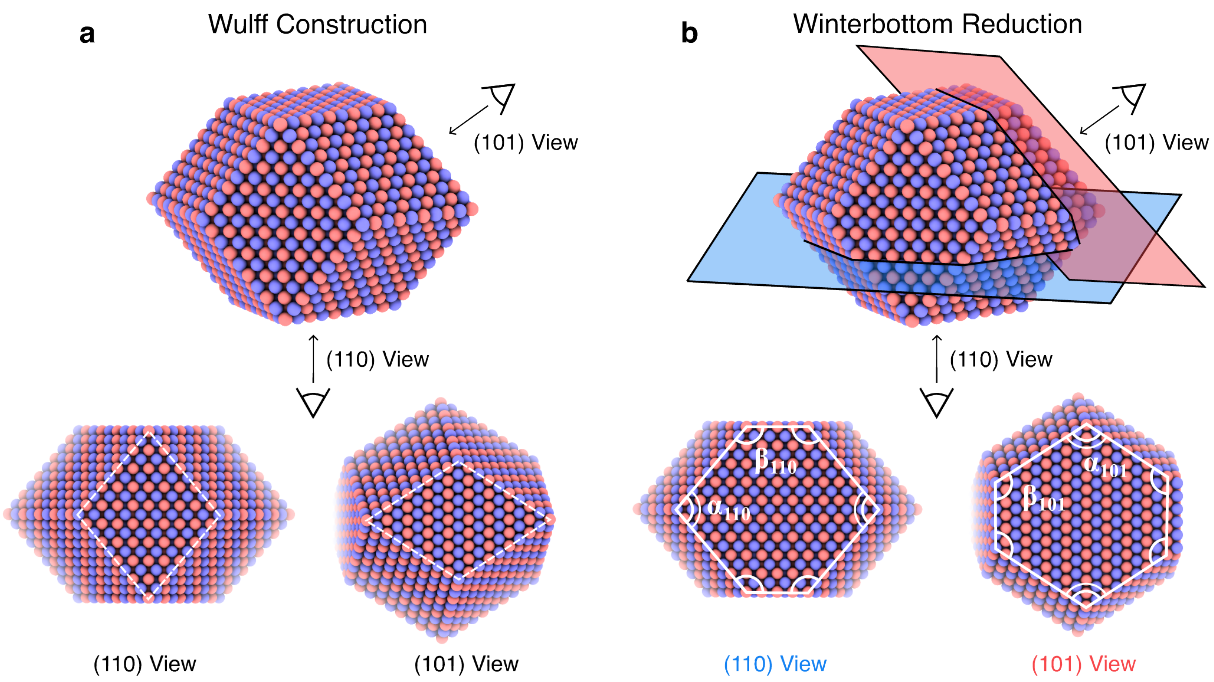

Supplementary Figure 11. **Schematic for facet growth at a surface.** (**a**) The Wulff construction for a BCT crystal with C=0.5. Below are shown the facets from the (110) and (101) planes. These show only four sides, highlighted by the white, dashed line. (**b**) A cartoon of the Winterbottom-like construction. Due to the presence of a surface, growth in one direction is inhibited. We model this by removing particles from a Wulff construction below a crystal plane, shown as blue for the (110) plane and red for the (101) plane. Below are the facets shown for these two planes. Due to the removal of particles, these facets now exhibit six sides with two distinct angles, α and β. The precise location where the plane cuts the Wulff constrution affects the edge lengths of the exposed facet, but the angles remain fixed.

Remarkably, despite the simplicity of the our approach, we find that the Winterbottom reduction captures the shape of our observed crystal facets, as well as their internal angles. In Suppl. Fig. 6a- 8a, the model crystal facets generated from the $G(r)$ fitting and Winterbottom reduction show the same facet angles as the experimental system. For the 600-nm, 430-nm, and 250-nm-diameter particles, we find that each particle size have an average C-value that describes the ensemble of measured structures. The corresponding Wulff constructions and Winterbottom reduction are shown in Suppl. Fig. 12, and the predicted facet angles for the (110) or (101) planes are shown in Suppl. Table 1. Importantly, the crystals synthesized by seeded growth also showed the same facet angles. Thus, we conclude that interactions with the coverslip can account for the observed differences between the expected equilibrium Wulff shapes and the observed (110) and (101) facets in our experiments.

d (nm)	α_{110}	β_{110}	α_{101}	β_{101}
250	109.5±0.4	125.3±0.4	109.5±0.4	125.3±0.4
430	106.2±1.6	126.9±0.8	111.1±0.8	124.4±0.4
600	97.0±1.9	131.5±1.0	116.0±1.1	122.0±0.5

Supplementary Table 1. **Expected facet angles.** Based on the average measured BCT structure for each particle size, this table lists the expected range of angles for (110) and (101) facets seen in experiment.

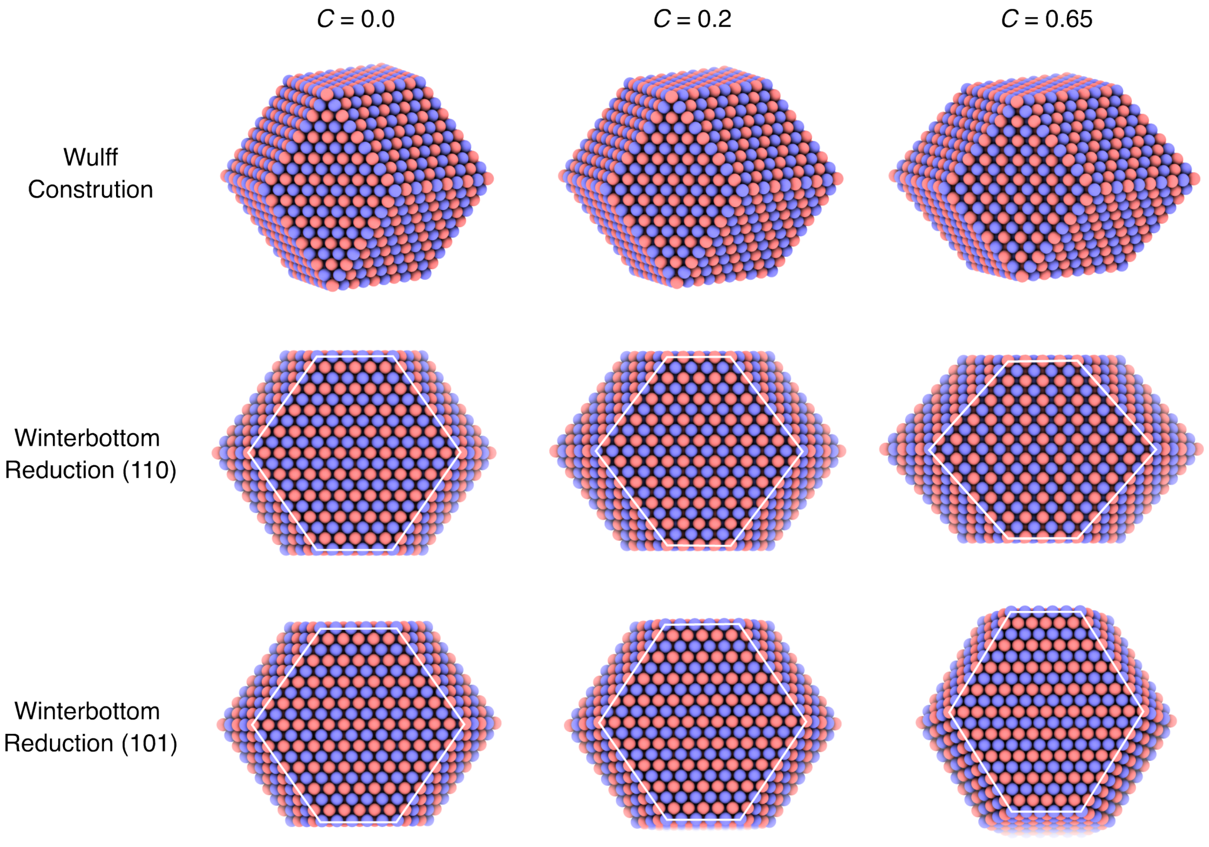

Supplementary Figure 12. **Models of expected crystal facets.** This shows the expected Wulff constructions and Winterbottom-like constructions of the (110) and (101) planes for the average C-values from the 250-nm (C=0), 430-nm (C=0.2) and 600-nm (C=0.65) crystals.

F. Rationalizing the observed structural colors

Fig. 4d,e in the main text shows examples of colloidal crystals assembled from 250-nm-diameter and 430-nm-diameter particles that show red and green coloration, respectively, in reflection under normal incidence. We rationalize the colors that we observe by considering Bragg diffraction from the crystalline lattices [13]. The wavelength in vacuum of the diffracted light should be approximately

$$\lambda = \frac{2n_c d_{(110)}}{n}, \tag{3}$$

where n_c is the effective refractive index of the crystal, n is the diffraction order, and $d_{(110)}$ is the distance between (110) planes of CsCl. For a perfect CsCl crystal, $d_{(110)} = 2\sqrt{2/3}r$ for a particle radius r.

We hypothesize that the 430-nm-diameter particle crystal shows green coloration, whereas the 250-nm-diameter particle crystal shows red, due to second-order diffraction. Assuming the red coloration of the 250-nm-diameter particle crystal is due to the primary ($n = 1$) Bragg reflection, we can estimate the effective refractive index of the colloidal crystal. Taking $\lambda \approx 650$ nm, we find a value of the effective refractive index of $n_c = 1.59$, which is approximately equal to the refractive index of polystyrene. This number seems reasonable given that roughly 70% of the crystal volume is composed of polystyrene. Substituting $n_c = 1.59$ and a particle radius $r = 430/2$ nm into our expression for the wavelength of diffracted light yields roughly $\lambda = 1100$ nm. While light of this color is squarely in the infrared, the second order diffracted light would have a wavelength of $\lambda = 550$ nm, which is in the green portion of the visible spectrum, as we observe in our experiments. Therefore, we conclude that the coloration of the 430-nm-diameter particle crystals is

likely due to second order diffraction, but this explanation would need to confirmed by direct experimental measurement to know definitively.

We also note that the coloration of the crystals in transmission is different from their coloration in reflection. For example, Fig. 3c shows snapshots during growth of a crystal of 250-nm-diameter particles imaged in transmission through crossed polarizers, which appears green. A crystal of the same type assembled from particles of the same size appears red when imaged in reflection (see Fig. 4e). As described above, we rationalize the color in reflection as arising from Bragg diffraction. However, the coloration in transmission is more complicated. For example, in an earlier publication [4], we showed that the coloration in transmission when imaged through crossed polarizers depends on the crystal orientation, as well as the orientation of the polarizers. In contrast, the coloration in reflection is independent of the presence or absence of the polarizers, as well as their orientation relative to the crystal orientation. This combination of differences makes interpretting structural coloration from transmission images more challenging and gives rise to the apparent difference of the green crystal in Fig. 3c (transmission) and the red crystal in Fig. 4e (reflection).

SUPPLEMENTARY NOTE 4. THEORETICAL ANALYSIS OF SELF-ASSEMBLY IN MICROFLUIDIC DROPLETS

In this section, we describe the theoretical model used to predict the probability of assembling a single-domain crystal within a droplet. We introduce the formal definition of τ_{g} and show how it can be calculated from a dynamical model of droplet-confined self-assembly based on classical theories of nucleation and growth. We then specialize the discussion to the case of discrete-step temperature-ramp experiments and show that the temperature step used in the experiments presented in the main text, $\Delta T = 0.1$ °C, is near optimal in this system.

A. Predicting the probability of single-crystal self-assembly in droplets

We first consider an arbitrary monotonic annealing protocol, $T(t)$. The probability that a single crystal assembles from an initial nucleation event occurring at temperature T is given by

$$p_{1\mathrm{x}}\big(T;T(t)\big) = p_{\text{first-nuc}}\big(T;T(t)\big) \times p_{\text{no-sec-nuc}}\big(T;T(t)\big). \tag{4}$$

The first term, $p_{\text{first-nuc}}$, is the probability that the initial nucleation event occurs at the temperature T and is given by

$$p_{\text{first-nuc}}\big(T;T(t)\big) = \left|\frac{dT}{dt}\right|^{-1} V_{\mathrm{drop}} k_{\mathrm{n}}\big(\rho_0, T\big) \exp\left[-\int_T^{T(0)} dT' \left|\frac{dT}{dt}\right|^{-1} V_{\mathrm{drop}} k_{\mathrm{n}}\big(\rho_0, T'\big)\right], \tag{5}$$

where V_{drop} is the droplet volume, k_{n} is the nucleation rate density, and ρ_0 is the initial uniform colloid concentration. In the case of an isothermal protocol, this reduces to $p_{\text{first-nuc}}(T) = 1 - \exp[-V_{\mathrm{drop}} k_{\mathrm{n}}(\rho_0, T) t_{\max}]$, where $t_{\max}$ is the duration of the experiment. If the annealing protocol begins above the melting temperature and ends at a temperature where there is no nucleation barrier, then the initial nucleation event must happen at some time during the protocol; this means that $p_{\text{first-nuc}}[T(t)] \equiv \int_{T(t_{\max})}^{T(0)} dT\, p_{\text{first-nuc}}(T;T(t)) = 1$. The second term, $p_{\text{no-sec-nuc}}$, is the probability that no subsequent nucleation events occur in the droplet. This probability depends on the growth dynamics of the crystal and its effect on the colloid concentration field, $\rho(r,t)$, elsewhere in the droplet. Assuming that the initial nucleation event occurs near the center of the droplet, we can approximate this term as

$$p_{\text{no-sec-nuc}}\big(T;T(t)\big) = \exp\left[-\int_{T(t_{\max})}^{T} dT' \left|\frac{dT}{dt}\right|^{-1} \int_{V_{\mathrm{drop}}} d\boldsymbol{r}\, k_{\mathrm{n}}\big(\rho(r,t), T'\big)\right]. \tag{6}$$

Finally, we can integrate over $T(t)$ to calculate $p_{1\mathrm{x}}$ for the complete protocol:

$$p_{1\mathrm{x}}[T(t)] \equiv \int_{T(t_{\max})}^{T(0)} dT\, p_{1\mathrm{x}}\big(T;T(t)\big) = \int_{T(t_{\max})}^{T(0)} dT\, p_{\text{first-nuc}}\big(T;T(t)\big) \times p_{\text{no-sec-nuc}}\big(T;T(t)\big). \tag{7}$$

Both $p_{\text{first-nuc}}$ and $p_{\text{no-sec-nuc}}$ are incredibly sensitive to the temperature due to their dependence on the nucleation rate density, k_{n}. It is therefore useful to introduce a characteristic growth time, τ_{g}, which represents the time required for all subsequent nucleation events to be suppressed. In the general case of a monotonic annealing protocol, we define τ_{g} according to

$$\int_0^{\tau_{\text{g}}} dt\, V_{\text{drop}} k_{\text{n}}\big(\rho_0, T(t_{\text{first-nuc}} + t)\big) = \int_{T(t_{\max})}^{T(t_{\text{first-nuc}})} dT' \left|\frac{dT}{dt}\right|^{-1} \int_{V_{\text{drop}}} d\boldsymbol{r}\, k_{\text{n}}\big(\rho(r,t), T'\big), \tag{8}$$

where $t_{\text{first-nuc}}$ is the time at which the initial nucleation event occurred. It turns out that τ_{g} varies slowly with $T_{\text{first-nuc}} \equiv T(t_{\text{first-nuc}})$, which allows for further approximations as discussed below. It is illuminating to consider the example of an isothermal protocol, in which case τ_{g} is given by

$$\tau_{\text{g}} = \int_0^{\infty} dt\, V_{\text{drop}}^{-1} \int_{V_{\text{drop}}} d\boldsymbol{r}\, \frac{k_{\text{n}}\big(\rho(r,t), T\big)}{k_{\text{n}}\big(\rho_0, T\big)} = \int_0^{\infty} dt \left\langle \frac{k_{\text{n}}\big(\rho(r,t), T\big)}{k_{\text{n}}(\rho_0, T)} \right\rangle_{V_{\text{drop}}}. \tag{9}$$

(The upper limit on the time integral can be extended to infinity if $\tau_{\text{g}} \ll t_{\max}$.) In this case, the ratio of nucleation rates, averaged over the droplet, decays to zero once equilibrium is established. The single-crystal probability can then be written as $p_{1\text{x}}(T) = \{1 - \exp[-V_{\text{drop}} k_{\text{n}}(\rho_0, T) t_{\max}]\} \exp[-V_{\text{drop}} k_{\text{n}}(\rho_0, T)\tau_{\text{g}}]$. In the more general case of a time-dependent protocol, τ_{g} has an analogous physical meaning, although the definition given above cannot be simplified in the same way. In what follows, we discuss how $\rho(r,t)$ can be calculated from the dynamical growth model, and we provide an illustrative example calculation for an isothermal protocol. We then demonstrate that τ_{g} is only weakly dependent on $T_{\text{first-nuc}}$, as claimed above.

B. Calculating τ_{g} from the dynamical growth model

We can calculate τ_{g} from the dynamical growth model presented in Ref. [4]. This model accounts for both reaction-limited nucleation kinetics, which are dependent on the rate at which DNA-grafted colloids roll or slide over one another, and diffusion-limited growth. Using the equilibrium and kinetic measurements performed in Ref. [4], we describe the crystal–vapor equilibrium and nucleation rate density by the equations

$$\log[\rho_{\text{eq}}(T)/\text{M}] = (10.50\ {}^\circ\text{C}^{-1})T - 573.39 \tag{10}$$

$$k_{\text{n}}(\rho, T) = (e^{22.4}\,\text{M}^{-1}\text{s}^{-1}/(10^{-13}\,\text{m}^3))\rho \exp[16\pi\gamma(T)^3/3(\log S)^2] \tag{11}$$

$$\gamma(T) = -(0.972\ {}^\circ\text{C}^{-1})T + 51.85, \tag{12}$$

where $\rho_{\text{eq}}(T)$ is the equilibrium colloidal gas density at temperature T, $S \equiv \rho/\rho_{\text{eq}}$ is the supersaturation, and $\gamma(T)$ is the surface tension of the crystal–vapor interface, multiplied by the colloid diameter squared. We then use the Wilson–Frenkel growth law, the continuity boundary condition at the crystal–vapor interface, and the conservation of colloids in the droplet to obtain the deterministic growth equations for the crystal radius, $R(t)$,

$$\frac{dR}{dt} = \frac{\alpha\alpha_{\text{diff}}}{\alpha + \alpha_{\text{diff}}} \left(\frac{v_\infty}{S}\right) \left[\sigma_\infty - 2\left(\frac{\xi}{R}\right)\right] \tag{13}$$

$$\sigma_\infty = \left[1 - \frac{3\alpha}{2(\alpha + \alpha_{\text{diff}})}\left(\frac{R}{R_{\text{drop}}}\right)\right]^{-1} \left[\frac{\rho_0 - \rho_{\text{eq}}}{\rho_{\text{eq}}} - \frac{\rho_{\text{c}}}{\rho_{\text{eq}}}\left(\frac{R}{R_{\text{drop}}}\right)^3 - \frac{3\alpha}{(\alpha + \alpha_{\text{diff}})}\left(\frac{\xi}{R_{\text{drop}}}\right)\right] \tag{14}$$

$$\alpha = \frac{\kappa/v_\infty}{1 + K(S=0)^{-1} + \kappa/v_\infty} \tag{15}$$

$$v_\infty = D\rho^{2/3}, \quad \alpha_{\text{diff}} = \frac{\rho_{\text{eq}} D S}{\rho_{\text{c}} v_\infty R}, \quad \rho_{\text{c}} = \frac{6\phi}{\pi d^3}, \quad \xi = \left(\frac{R^*}{R}\right)^2 \gamma, \quad R^* = \frac{2\gamma\rho_{\text{eq}}}{\rho_0 - \rho_{\text{eq}}}, \tag{16}$$

with constants

$$D = 10^{-12}\,\text{m}^2\text{s}^{-1}, \quad d = 6 \times 10^{-7}\,\text{m}, \quad \phi = 0.74, \quad \kappa = 1\,\text{s}^{-1}, \quad \text{and} \quad K(S=0) = 0.01. \tag{17}$$

Here, ρ_0 is the initial colloid number density, D is the self-diffusion coefficient, ϕ is the packing fraction in the crystal phase, and κ is the typical time required for a colloid to diffuse its own diameter while rolling or sliding on another colloid. The equilibrium constant K describes the attachment of a single colloid to a growing nucleus, which gives rise to an attachment coefficient, α, that is less than unity at low supersaturations, as described in Ref. [4]. Note that we have modified the Gibbs–Thomson term involving ξ such that it decays rapidly once the crystal grows larger than R^*; this modification is necessary for the vapor phase in the finite-size droplet to reach equilibrium and can be justified by noting that the crystal rapidly develops planar interfaces beyond the critical nucleus size. Also note that if the annealing protocol is time-dependent, then the quantities $\rho_{\rm eq}$ and γ are time-dependent as well.

We solve the differential equation for the crystal radius numerically using the Euler method starting from an initial radius 10% larger than the critical radius, R^*. We then compute the time-dependent concentration field, $\rho(r,t)$, from the numerical solution to the differential equation for $R(t)$,

$$\sigma(r,t) \equiv \frac{\rho(r,t)-\rho_{\rm eq}}{\rho_{\rm eq}} = \sigma_\infty - \frac{\alpha}{\alpha+\alpha_{\rm diff}}\left[\sigma_\infty - 2\left(\frac{\xi}{R}\right)\right]\left(\frac{R}{r}\right), \quad R < r < R_{\rm drop}. \tag{18}$$

With expressions for $\rho(r,t)$ and $k_{\rm n}(\rho,T)$, we can evaluate $\tau_{\rm g}$ using the definition given in Eq. (8). Example calculations of the time-dependent concentration field and $\tau_{\rm g}$ for an isothermal protocol are shown in Suppl. Fig. 13. Importantly, this example shows that the average nucleation rate across the entire droplet does not decrease substantially from its initial value until the concentration profile spreads out to reach the edge of the droplet at $r = R_{\rm drop}$. Thus, at times shorter than $\tau_{\rm g}$, regions within the droplet volume that are far from the initial nucleation site do not feel any effects of the growing crystal. We also show that $\tau_{\rm g}$ is indeed weakly dependent on the temperature, despite $k_{\rm n}$ varying by orders of magnitude over a temperature range of ~ 0.1 °C, in Suppl. Fig. 14.

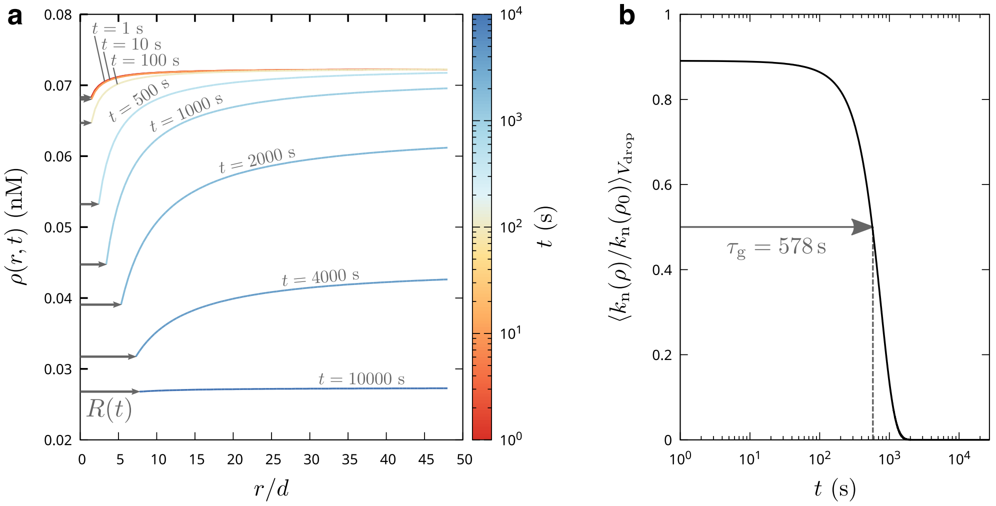

Supplementary Figure 13. **Post-nucleation effects on the particle concentration profile in a droplet.** (**a**) An example concentration profile measured from the center of the droplet as a function of the time post nucleation [i.e., $R(t=0) = 1.1R^*$] in an isothermal protocol [$T = 52.3$ °C, $V_{\rm drop} = 10^{-13}\,{\rm m}^3$, and $\rho_0 = 0.5\%\,({\rm v/v})$]. The inner boundary condition for the concentration profile is located at the crystal–vapor interface, $R(t)$. (**b**) The corresponding calculation of $\tau_{\rm g}$. Note that the ratio of nucleation rates starts below unity since, according to the deterministic growth model, the critical nucleus itself is sufficient to establish a non-uniform concentration field. Comparison of panels (**a**) and (**b**) shows that the average nucleation rate across the droplet volume does not decrease substantially until the concentration profile spreads out across the entire droplet and the outer boundary condition begins to decrease from its initial value, ρ_0.

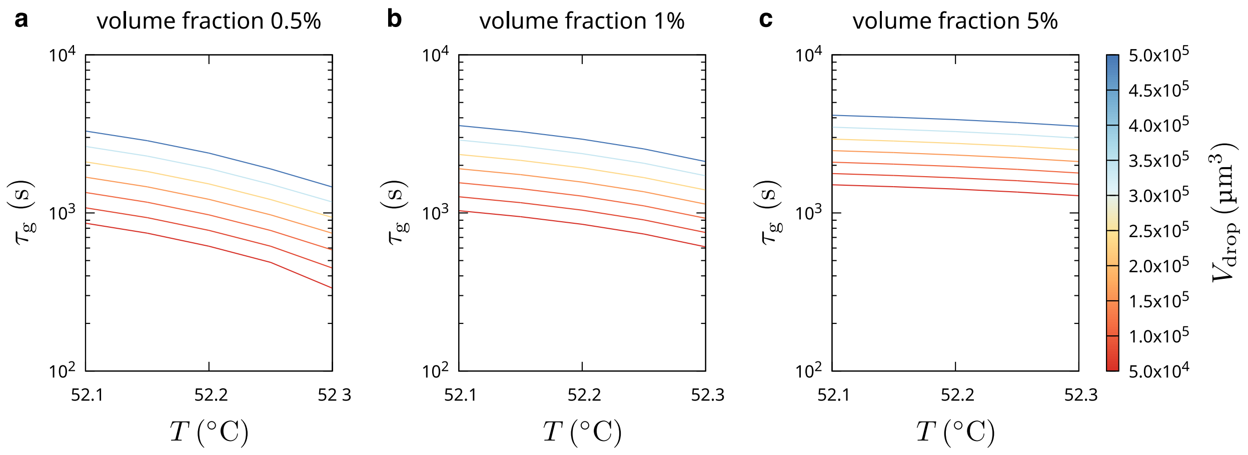

Supplementary Figure 14. **Volume fraction dependence of the growth time, τ_g.** Compared to k_n, which varies by orders of magnitude over a 0.1 °C temperature range, calculations using the deterministic growth model show that τ_g is relatively insensitive to temperature. Panels show example calculations assuming an initial particle volume fraction of (**a**) 0.5%, (**b**) 1%, and (**c**) 5%.

C. Predicting the probability of single-crystal self-assembly in discrete-step temperature-ramp droplet experiments

We now apply the theory developed above to predict the probability of assembling a single crystal in a discrete-step temperature-ramp experiment. Since we do not know the exact temperature at which the protocol is initiated [i.e., $T(0)$] in any particular experimental run, we approximate the expected value of $p_{1x}[T(t)]$ by assuming a continuous linear ramp with constant ramp rate dT/dt. The probability that the initial nucleation event occurs at the temperature T in this approximation is thus

$$p_{\text{first-nuc}}\big(T;T(t)\big) = \left|\frac{dT}{dt}\right|^{-1} V_{\text{drop}} k_{\text{n}}(\rho_0,T) \exp\left[-\left|\frac{dT}{dt}\right|^{-1}\int_T^\infty dT'\, V_{\text{drop}} k_{\text{n}}(\rho_0,T')\right]. \quad (19)$$

However, it is crucial that the computation of $p_{\text{no-sec-nuc}}$ captures the stepwise nature of the protocol, since each step of the protocol is essentially isothermal over a time period, Δt, that is much longer than τ_g. We therefore compute the probability of a second nucleation event using the isothermal expression under the assumption that $\tau_g \ll \Delta t$,

$$p_{\text{no-sec-nuc}}\big(T;T(t)\big) = \exp[-V_{\text{drop}} k_{\text{n}}(\rho_0,T)\tau_{\text{g}}(T)]. \quad (20)$$

We can now take advantage of the weak temperature dependence of τ_g by solving the growth model only at the most probable initial nucleation temperature. We do so by computing the expected value of the initial nucleation temperature,

$$\langle T_{\text{first-nuc}}\rangle_{T(t)} = \int_0^\infty dT\, T p_{\text{first-nuc}}(T;T(t)), \quad (21)$$

and then evaluating τ_g^* at $\langle T_{\text{first-nuc}}\rangle_{T(t)}$ using the isothermal expression, Eq. (9). The final expression for the single crystal probability is

$$p_{1x}[T(t)] = \left|\frac{dT}{dt}\right|^{-1}\int_0^\infty dT\, V_{\text{drop}} k_{\text{n}}(\rho_0,T) \exp\left[-\left|\frac{dT}{dt}\right|^{-1}\int_T^\infty dT'\, V_{\text{drop}} k_{\text{n}}(\rho_0,T') - V_{\text{drop}} k_{\text{n}}(\rho_0,T)\tau_{\text{g}}^*\right]. \quad (22)$$

Fig. 2 in the main text shows the comparison between the predictions made using this approach and the results of discrete-step temperature-ramp experiments. Importantly, the predictions do not involve any adjustable parameters. The overestimation of p_{1x} at the slowest ramp rates is likely due to partial evaporation of the droplets, which tends to increase the supersaturation more quickly than the rate specified by the

temperature protocol alone. The fact that this effect is greatest in the smallest droplets is consistent with this hypothesis, since the initial nucleation event is expected to occur at lower temperatures (and thus later times in a protocol) in these cases.

D. Comparing the probability of secondary nucleation events in discrete-step and continuous temperature-ramp droplet experiments

This theory predicts that the stepwise nature of the temperature ramps used in our experiments substantially increases the probability of assembling a single crystal. For the sake of comparison, we can consider what happens when the temperature ramp is continuous. In this case, we must use Eq. (8) to compute τ_g^*, assuming that the initial nucleation event occurs at $\langle T_{\text{first-nuc}} \rangle_{T(t)}$ and that the temperature continues to decrease linearly in time after $t_{\text{first-nuc}}$. Note that it is still reasonable to use τ_g^* instead of computing $\tau_g(T)$, since τ_g remains weakly dependent on the initial nucleation temperature in the case of a continuous temperature ramp. The probability of assembling a single crystal using such a protocol is thus

$$p_{1x}[T(t)] = \left|\frac{dT}{dt}\right|^{-1} \int_0^\infty dT\, V_{\text{drop}} k_n(\rho_0, T) \exp\left[-\left|\frac{dT}{dt}\right|^{-1} \int_{T-|dT/dt|\tau_g^*}^{\infty} dT'\, V_{\text{drop}} k_n(\rho_0, T')\right]. \quad (23)$$

Suppl. Fig. 15 shows that p_{1x} is always predicted to be lower using a continuous protocol than it is using a stepwise protocol with the same average ramp rate, assuming that $\tau_g \ll \Delta t$. Furthermore, the relative performance of the continuous protocol decreases as the droplet volume and the ramp rate increase. These effects can be understood by considering the ratio of the τ_g^* values for the continuous and stepwise protocols, which is always greater than unity. In the case where the temperature continues to decrease continuously following the initial nucleation event, the homogeneous nucleation rate far from the initial nucleus actually *increases* before the nonuniform concentration profile is able to spread out across the entire droplet via diffusion. The net result is that it takes longer in a continuous protocol for the growth of the first crystal to suppress nucleation elsewhere in the droplet, particularly when the droplet is large and/or the ramp rate is fast.

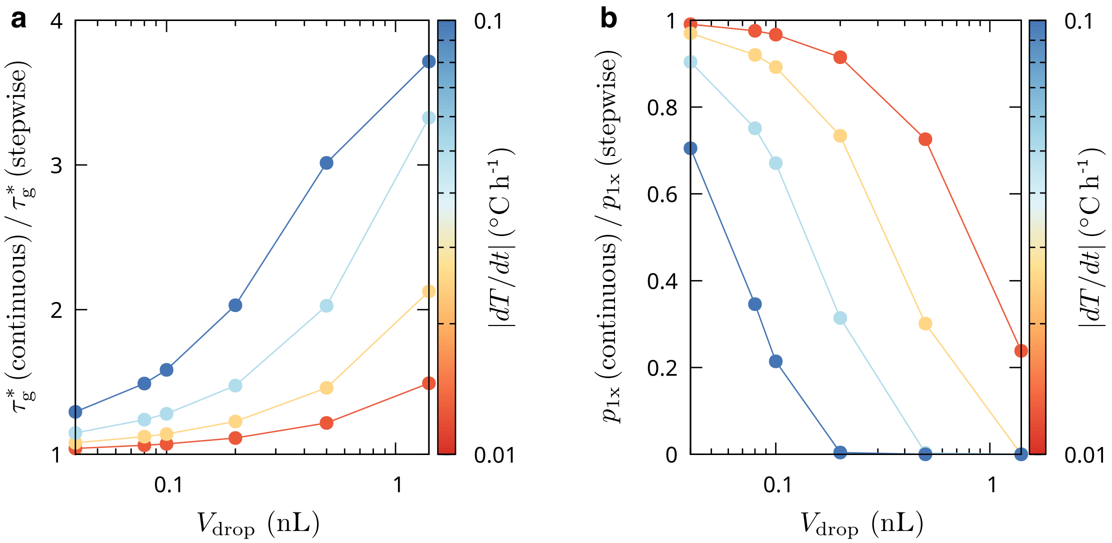

Supplementary Figure 15. **Comparison between discrete and continuous temperature ramps.** Comparison of (**a**) τ_g^* and (**b**) the predicted single crystal fraction resulting from continuous versus stepwise temperature ramps with the same average ramp rate, dT/dt, assuming an initial colloid volume fraction of 0.5%.

E. Predicting an optimal temperature-ramp protocol

The analysis above shows that discrete steps of duration $\tau_g \ll \Delta t$ tend to increase $p_{\text{no-sec-nuc}}$ relative to a continuous protocol. However, this theory cannot hold for all protocols with discrete steps of arbitrary duration. On the one hand, a continuous protocol can be represented in practice by a discrete protocol with the step height, ΔT, and width, Δt, made extremely small. On the other hand, a protocol with arbitrarily large ΔT is essentially a deep quench, which we know does not lead to single-crystal assembly. This raises the question of what the optimal step height should be when designing a discrete-step temperature-ramp protocol at a fixed average ramp rate.

To address this question, we revisit the key assumptions made in developing the theory for the discrete-step protocol in Suppl. Note 3C. It is important to note that the absolute temperature at which the ramp starts, $T(0)$, is a relevant parameter; in other words, we need to specify ΔT, $|dT/dt| = |\Delta T/\Delta t|$, and $T(0)$ to define a discrete-step temperature ramp precisely. However, because we typically do not know $T(0)$ to sufficient accuracy in an experiment, it is only practical to predict a range of possible outcomes for protocols whose initial temperature is randomly selected from a uniform distribution. This is the strategy taken in the calculations below.

First, we consider the accuracy of the continuous approximation for $p_{\text{first-nuc}}$, Eq. (19), used in the comparison between discrete-step and continuous ramps above. Suppl. Fig. 16a shows the predicted temperatures at which the initial nucleation event occurs in discrete and continuous protocols as a function of ΔT for selected ramp rates. In the case of the predictions for discrete protocols, the points and error bars indicate the mean and standard deviation, respectively, of $\langle T_{\text{first-nuc}}\rangle$ for a uniform distribution of starting temperatures, $T(0) \in [T_0, T_0 + \Delta T)$, where T_0 is a temperature above the melting temperature. The initial nucleation temperatures tend to be lower when using discrete protocols, although the variations resulting from differences in $T(0)$ can be quite large. This comparison indicates that the continuous approximation, Eq. (19), is likely to be sufficiently accurate [i.e., within the uncertainty due to $T(0)$] for step heights up to approximately 0.1 °C. In this range of $\Delta T \lesssim 0.1$ °C, we find that τ_g^*, which is computed at the average expected first-nucleation temperature $\langle T_{\text{first-nuc}}\rangle$, is indeed greater for discrete protocols than for continuous protocols, as shown in Suppl. Fig. 16b. However, the values of τ_g^* for the discrete protocols tend to decrease at large step heights due to the decrease in the mean $\langle T_{\text{first-nuc}}\rangle$.

Second, we consider the suitability of the isothermal approximation for $p_{\text{no-sec-nuc}}$ used in Eq. (22). This approximation is only valid when $\tau_g^* \ll \Delta t$. As shown in Suppl. Fig. 16c, this condition is met when ΔT is large and the ramp rate is slow. This approximation is thus likely to be of sufficient accuracy for step heights larger than about 0.05 °C.

Taken together, the example calculations reported in Suppl. Fig. 16 indicate that Eq. (22), which treats the initial nucleation probability using a continuous temperature ramp and the secondary nucleation probability using an isothermal protocol, is applicable only if the temperature step height is on the order of 0.05 to 0.1 °C. If the step height is smaller than this range (i.e., the protocol is closer to being continuous), then crystal growth cannot be considered to be isothermal, and probability of secondary nucleation increases. Yet if the step height is larger than this range (i.e., the protocol is less continuous), then the initial nucleation event is more likely to occur at a low temperature, which implies a faster initial nucleation rate and thus also promotes secondary nucleation. Thus, in addition to Eq. (22) being most applicable in this range of step heights, we also expect these stepwise protocols to yield single crystals with the highest probability given an imposed ramp rate (or, equivalently, a prescribed maximum duration of the experiment). Nonetheless, we should keep in mind that there is more inherent variability in the results of discrete-step temperature-ramp protocols due to the uncertainty in the absolute starting temperature, $T(0)$, which means that the actual yield may vary slightly from experiment to experiment.

SUPPLEMENTARY NOTE 5. THEORETICAL ANALYSIS OF SEEDED GROWTH EXPERIMENTS

A. Identifying isolated seeds and diffusion-limited growth

Seeded growth experiments are carried out using monodisperse, pre-assembled seeds in a bath of 'weak' colloidal particles at constant supersaturation. The annealing conditions are chosen such that the weak particles are only moderately supersaturated, leading to growth of the crystalline seeds without homogeneous

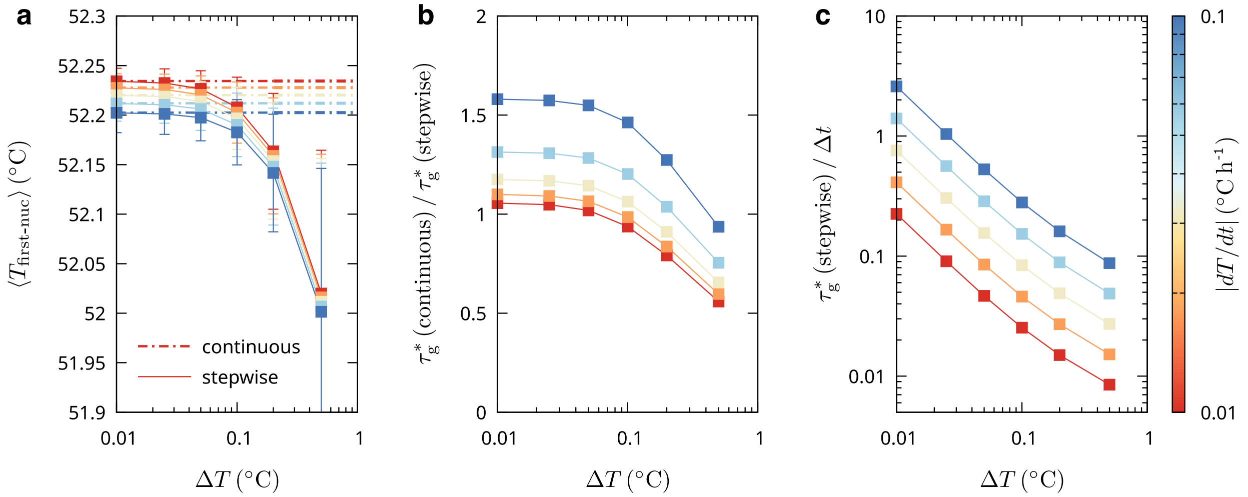

Supplementary Figure 16. **Growth from continuous or step-wise temperature ramps.** Comparisons of (**a**) first-nucleation temperatures and (b) $\tau_{\rm g}^*$ for continuous and stepwise protocols with varying step heights, ΔT. The error bars indicate the standard deviation assuming a uniform distribution of starting temperatures. (**c**) Comparison of $\tau_{\rm g}^*$ to the step width, Δt, for varying step heights and ramp rates.

nucleation of new crystals from the weak-particle bath. Analogously to our discussion of late-stage crystal growth within droplets, we expect the growth of isolated seeds in bulk solution under these conditions to be diffusion-limited. Starting from an initial spherical seed containing N_0 particles, we integrate Eq. (13) in the limit $\alpha \gg \alpha_{\rm diff}$ and $R \gg \xi$ to obtain the deterministic diffusion-limited growth law

$$N(t) = \left\{ \frac{2}{3} \left[\frac{3}{2} N_0^{2/3} + \frac{2\pi d D}{\phi^{1/3}} \left(\rho_0 - \rho_{\rm eq} \right) t \right] \right\}^{3/2}, \tag{24}$$

where ρ_0 and $\rho_{\rm eq}$ are the initial and equilibrium concentrations, respectively, of the weak particles in the bulk phase, and time is measured from the beginning of the seeded growth experiment. At long times, when $N(t) \gg N_0$, this expression predicts the scaling law $N(t) \sim t^{3/2}$.

Fig. 3b in the main text shows that the scaling law predicted for diffusion-limited growth at constant supersaturation is borne out by the data. However, we find that the prefactor dictating the specific rate of the growth can decrease if the seeds are too close together. To understand how far apart seeds must be placed in order to grow at the maximum rate, we consider the concentration profile predicted by Eq. (18). In the limit $\alpha \gg \alpha_{\rm diff}$ and $R \gg \xi$ relevant to growth in the weak-particle bath, the decrease in the local particle concentration due to the growth of a single isolated crystal decays inversely with the ratio $r/R(t)$, where r is the distance from the center of the crystal and $R(t)$ is the current radius of the crystal. We therefore predict that a crystal will be effectively isolated for the entirety of the seeded growth experiment as long as it is separated from all other seeds by a distance of at least $3R(t_{\rm max})$, where $t_{\rm max}$ is the duration of the experiment. This choice ensures that the far-field reduced chemical potential difference, $\Delta\mu/{\rm k_B}T \simeq S - 1$, of crystals identified as being isolated never drops below $2/3$ of the bulk value. As demonstrated in Fig. 3b of the main text, this criterion for identifying effectively isolated seeds allows us to select crystals that maintain a monodisperse size distribution during seeded growth. This criterion is also illustrated in Suppl. Fig. 17, which shows a mixture of isolated and non-isolated crystals within the same field of view.

B. Comparison with isothermal nucleation and growth in bulk solution

To emphasize the surprisingly narrow size distribution of isolated crystals grown using our two-step protocol, we compare our final crystal sizes with the distribution of crystal sizes obtained from an isothermal experiment in the inset of the main text Fig. 3b. This bulk-nucleation experiment was analyzed at a time when some crystals were roughly the size as the isolated crystals from the seeded experiment; the image

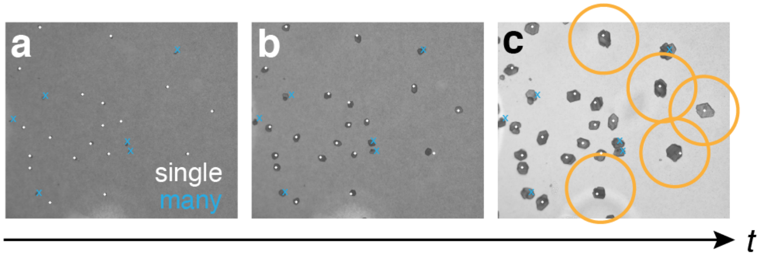

Supplementary Figure 17. **Isolated crystal growth.** In seeded growth experiments, a high local concentration of seeds can reduce the growth rate of nearby seeds. (**a**) We first distinguish between single-crystal seeds (white dots) and polycrystal seeds (blue crosses). (b–c) Crystals grown from single-crystal seeds are then classified as being isolated if the initial seed is separated from all other seeds by a distance of at least 3 times the diameter of the crystal at the conclusion of the seeded growth experiment, as indicated by the yellow circles on the right. The isolated crystals are also the largest crystals in the final frame, indicating that they grew the fastest as described in the text.

used in this analysis is shown in Suppl. Fig. 18. The differences between the bulk and seeded nucleation experiments can be understood by noting that uniform supersaturation of a bulk fluid leads to stochastic nucleation. Because the nucleation times are exponentially distributed, the time periods over which the various crystals can grow vary widely. The resulting distribution of final crystal sizes is therefore extremely broad. The formation of polycrystals is also apparent in Suppl. Fig. 18, since the initial nucleation events also occur randomly in space.

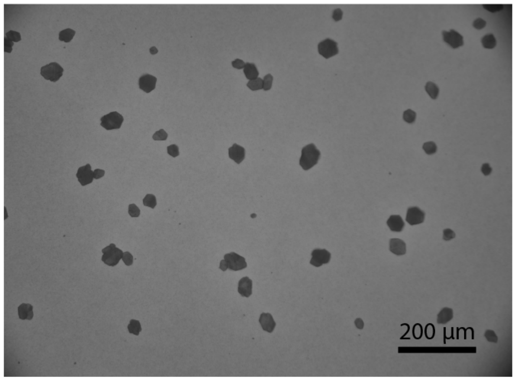

Supplementary Figure 18. **Isothermal crystal nucleation and growth.** An image of crystals formed by isothermal nucleation and growth in bulk that was compared to the data from the seeded growth experiment in Fig. 3b of the main text.

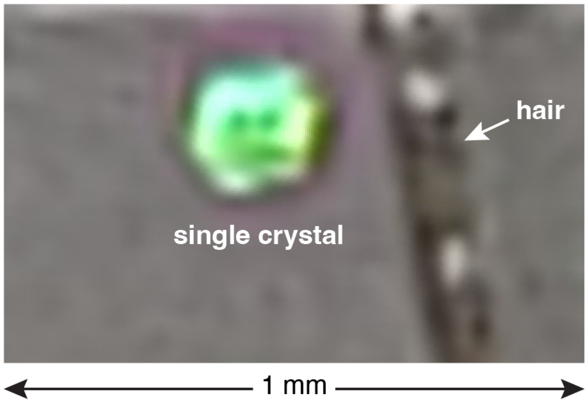

Supplementary Figure 19. **A macroscopic single crystal that can be seen by the naked eye.** A cellphone image of a capillary containing a large single crystal formed from 400-nm-diameter particles next to a human hair taken without any intermediate optics. The width of the image is 1 mm.

Particle Size (nm)	Lattice Spacing (nm)	Crystal Length (µm)	Particle Number	Reference
5	25.7^{SAXS}	2.2	6.3×10^5	[9]
5	15.3^{SAXS}	3.5	1.2×10^7	[14]
5	28^{SAXS}	62	1.1×10^{10}	[15]
10	29.1^{SAXS}	3.56	1.8×10^6	[9]
**10**	31.7^{SAXS}	70.5	1.1×10^{10}	[15]
14	20.3^{SEM}	2.1	1.1×10^6	[16]
15	35.5^{SAXS}	1.2	3.9×10^4	[17]
15	15.9^{SEM}	1.9	1.7×10^6	[18]
15	27^{SAXS}	7.3x2.4x2.4	2.4×10^6	[19]
**15**	15.6^{SEM}	2.4	3.6×10^6	[20]
20	52^{SAXS}	2.7	1.4×10^5	[21]
20	31.4^{SEM}	2.1	3.0×10^5	[22]
20	56^{SEM}	4.8	6.3×10^5	[12]
**20**	24^{SEM}	2.9	1.8×10^6	[9]
30	31^{SEM}	2.5	5.2×10^5	[23]
40	46.7^{SEM}	1.2	1.7×10^4	[24]
**59.6**	75.5^{SEM}	6.5	6.4×10^5	[25]
400		6.8	4.9×10^3	[26]
400		12.5	3.1×10^4	[27]
**400**		22	1.6×10^5	[4]
**600**		28.5	1.1×10^5	[4]
1000		16	4.1×10^3	[28]
1000		22.7	1.2×10^4	[29]
1000		26.5	1.9×10^4	[30]
1000		32.9	3.6×10^4	[31]
1000		36.4	4.8×10^4	[32]
**1000**		39.1	6.0×10^4	[33]

Supplementary Table 2. **Review of the largest reported crystal sizes in the literature.** For particles with size less than 100 nm, we used the lattice spacing of the crystals to get a better estimate of the number of particles; when available, we used provided SAXS data for this estimate, otherwise, distances between neighboring particles were measured (see Suppl. Note 2B). For Ref. [19], the crystals formed hexagonal prisms, so the dimensions of an estimated bounding rectangular prism are listed. Entries with bolded particle sizes are the largest reported structures for that relative size of particle.

SUPPLEMENTARY REFERENCES

[1] S. Whitelam and R. L. Jack, The statistical mechanics of dynamic pathways to self-assembly, Annual review of physical chemistry **66**, 143 (2015).

[2] V. J. Anderson and H. N. Lekkerkerker, Insights into phase transition kinetics from colloid science, Nature **416**, 811 (2002).

[3] P. R. t. Wolde and D. Frenkel, Enhancement of protein crystal nucleation by critical density fluctuations, Science **277**, 1975 (1997).

[4] A. Hensley, W. M. Jacobs, and W. B. Rogers, Self-assembly of photonic crystals by controlling the nucleation and growth of dna-coated colloids, Proceedings of the National Academy of Sciences **119**, e2114050118 (2022).

[5] J. C. Crocker and D. G. Grier, Methods of digital video microscopy for colloidal studies, Journal of Colloid and Interface Science **179**, 298 (1996).

[6] D. Chandler, *Introduction to Modern Statistical Mechanics* (Oxford University Press, 1987).

[7] F. Cui, S. Marbach, J. A. Zheng, M. Holmes-Cerfon, and D. J. Pine, Comprehensive view of microscopic interactions between dna-coated colloids, Nature Communications **13**, 1 (2022).

[8] G. Wulff, Xxv. zur frage der geschwindigkeit des wachsthums und der auflösung der krystallflächen, Zeitschrift für Kristallographie-Crystalline Materials **34**, 449 (1901).

[9] E. Auyeung, T. I. Li, A. J. Senesi, A. L. Schmucker, B. C. Pals, M. O. de La Cruz, and C. A. Mirkin, DNA-mediated nanoparticle crystallization into wulff polyhedra, Nature **505**, 73 (2014).

[10] M. N. O'Brien, H.-X. Lin, M. Girard, M. Olvera de la Cruz, and C. A. Mirkin, Programming colloidal crystal habit with anisotropic nanoparticle building blocks and dna bonds, Journal of the American Chemical Society **138**, 14562 (2016).

[11] W. L. Winterbottom, Equilibrium shape of a small particle in contact with a foreign substrate, Acta Metallurgica **15**, 303 (1967).

[12] D. J. Lewis, L. Z. Zornberg, D. J. Carter, and R. J. Macfarlane, Single-crystal winterbottom constructions of nanoparticle superlattices, Nature Materials **19**, 719 (2020).

[13] S. A. Asher, J. M. Weissman, A. Tikhonov, R. D. Coalson, and R. Kesavamoorthy, Diffraction in crystalline colloidal-array photonic crystals, Physical Review E **69**, 066619 (2004).

[14] E. Auyeung, W. Morris, J. E. Mondloch, J. T. Hupp, O. K. Farha, and C. A. Mirkin, Controlling structure and porosity in catalytic nanoparticle superlattices with dna, Journal of the American Chemical Society **137**, 1658 (2015).

[15] S. Lee, H. A. Calcaterra, S. Lee, W. Hadibrata, B. Lee, E. Oh, K. Aydin, S. C. Glotzer, and C. A. Mirkin, Shape memory in self-adapting colloidal crystals, Nature **610**, 674 (2022).

[16] A. F. De Fazio, A. H. El-Sagheer, J. S. Kahn, I. Nandhakumar, M. R. Burton, T. Brown, O. L. Muskens, O. Gang, and A. G. Kanaras, Light-induced reversible dna ligation of gold nanoparticle superlattices, ACS Nano **13**, 5771 (2019).

[17] J. Zhu, H. Lin, Y. Kim, M. Yang, K. Skakuj, J. S. Du, B. Lee, G. C. Schatz, R. P. Van Duyne, and C. A. Mirkin, Light-responsive colloidal crystals engineered with dna, Advanced Materials **32**, 1906600 (2020).

[18] S. E. Seo, M. X. Wang, C. M. Shade, J. L. Rouge, K. A. Brown, and C. A. Mirkin, Modulating the bond strength of dna–nanoparticle superlattices, ACS Nano **10**, 1771 (2016).

[19] S. E. Seo, M. Girard, M. Olvera de la Cruz, and C. A. Mirkin, Non-equilibrium anisotropic colloidal single crystal growth with dna, Nature Communications **9**, 4558 (2018).

[20] S. Lee, C. Y. Zheng, K. E. Bujold, and C. A. Mirkin, A cross-linking approach to stabilizing stimuli-responsive colloidal crystals engineered with DNA, J. Am. Chem. Soc. **141**, 11827 (2019).

[21] D. J. Park, J. C. Ku, L. Sun, C. M. Lethiec, N. P. Stern, G. C. Schatz, and C. A. Mirkin, Directional emission from dye-functionalized plasmonic dna superlattice microcavities, Proceedings of the National Academy of Sciences **114**, 457 (2017).

[22] T. Oh, S. S. Park, and C. A. Mirkin, Stabilization of colloidal crystals engineered with dna, Advanced Materials **31**, 1805480 (2019).

[23] E. Auyeung, R. J. Macfarlane, C. H. J. Choi, J. I. Cutler, and C. A. Mirkin, Transitioning dna-engineered nanoparticle superlattices from solution to the solid state, Advanced Materials **24**, 5181 (2012).

[24] S. Wang, S. S. Park, C. T. Buru, H. Lin, P.-C. Chen, E. W. Roth, O. K. Farha, and C. A. Mirkin, Colloidal crystal engineering with metal–organic framework nanoparticles and dna, Nature communications **11**, 2495 (2020).

[25] A. J. Senesi, D. J. Eichelsdoerfer, K. A. Brown, B. Lee, E. Auyeung, C. H. J. Choi, R. J. Macfarlane, K. L. Young, and C. A. Mirkin, Oligonucleotide flexibility dictates crystal quality in dna-programmable nanoparticle superlattices, Advanced Materials **26**, 7235 (2014).

[26] M. T. Casey, R. T. Scarlett, W. Benjamin Rogers, I. Jenkins, T. Sinno, and J. C. Crocker, Driving diffusionless transformations in colloidal crystals using DNA handshaking, Nature Communications **3**, 1209 (2012).

[27] Y. Wang, I. C. Jenkins, J. T. McGinley, T. Sinno, and J. C. Crocker, Colloidal crystals with diamond symmetry at optical lengthscales, Nature Communications **8**, 14173 (2017).

[28] W. B. Rogers and V. N. Manoharan, Programming colloidal phase transitions with DNA strand displacement, Science **347**, 639 (2015).

[29] Y. Wang, Y. Wang, X. Zheng, É. Ducrot, M.-G. Lee, G.-R. Yi, M. Weck, and D. J. Pine, Synthetic strategies toward dna-coated colloids that crystallize, Journal of the American Chemical Society **137**, 10760 (2015).

[30] J. Lowensohn, A. Hensley, M. Perlow-Zelman, and W. B. Rogers, Self-assembly and crystallization of DNA-coated colloids via linker-encoded interactions, Langmuir **36**, 7100 (2020).

[31] J. S. Oh, M. He, G.-R. Yi, and D. J. Pine, High-density dna coatings on carboxylated colloids by dmtmm-and azide-mediated coupling reactions, Langmuir **36**, 3583 (2020).

[32] Y. Wang, Y. Wang, X. Zheng, É. Ducrot, J. S. Yodh, M. Weck, and D. J. Pine, Crystallization of DNA-coated colloids, Nature Communications **6**, 7253 (2015).

[33] J. S. Oh, G.-R. Yi, and D. J. Pine, Reconfigurable self-assembly and kinetic control of multiprogrammed dna-coated particles, ACS Nano **14**, 4595 (2020).
